# Supplementary material for: Characterizing physical activity bouts in people with stroke with different ambulation statuses
Source: PLoS One. 2025 May 14;20(5):e0307625. doi: 10.1371/journal.pone.0307625 (PMC12077672; doi:10.1371/journal.pone.0307625)
Supplement: Attachment — Submitted filename: Response letter.docx [file pone.0307625.s002.docx]

Dear Editor,

Thank you for the opportunity to re-submit this appeal regarding our manuscript titled ‘*Characterizing physical activity bouts in people with stroke with different ambulation statuses*’.

Below is our point-by-point response to the issues raised by the two external reviewers. We have also attached a revised manuscript with the changes highlighted.

**Reviewer 1**

The paper investigates how physical activity (PA) varies between stroke survivors with differing ambulatory capabilities and compares them with healthy controls. Using accelerometers, the study characterizes and contrasts daily PA patterns, frequencies, and intensities among limited community ambulators, community ambulators, and healthy controls. It has several strengths:
• The paper addresses an important gap in the literature by exploring the PA patterns in post-stroke individuals with varying ambulation statuses, providing a nuanced understanding of their activity profiles.
• The use of accelerometers (Actigraph GT3X+) for objective measurement of PA provides accurate and reliable data, enhancing the credibility of the findings.
• The paper provides clear and practical recommendations for improving cardiovascular health in stroke survivors, emphasizing tailored interventions based on ambulation status.
• Ethical considerations were well-addressed, including approval from the Regional Board of Ethics in Stockholm and informed consent from participants.

However, it has a few major weaknesses:

**Reviewer comment 1:** Small Sample Size: The study only includes 39 participants post-stroke, limiting the generalisability of the findings to a broader population of stroke survivors.
**Authors response:** We appreciate the reviewer for raising this concern. While we acknowledge that the sample size is relatively small, which indeed limits the generalizability of the findings—a point we have already highlighted as a study limitation in the manuscript—we believe that this study offers valuable insights into the physical activity behavior of individuals post-stroke with varying ambulation statuses. This is particularly crucial for limited community ambulators, who are especially vulnerable to secondary health complications resulting from physical inactivity.

**Reviewer comment 2:** Limited Diversity in Participants: The study's participants are predominantly younger and have fewer women than the typical stroke population in Sweden, potentially affecting the applicability of the findings.

**Authors response:** We appreciate the reviewer’s comment regarding the limited diversity among the study participants. We acknowledge that the demographic imbalance is a limitation, and this has been noted in our manuscript. Despite this limitation, as mentioned in our response to Comment 1, we believe the study provides valuable insights into the physical activity patterns across different times of the day in people post-stroke with varying ambulation statuses. While the demographics may not fully represent the broader stroke population in Sweden, the findings still offer critical information that can inform interventions for specific subgroups, such as younger stroke survivors with different ambulation statuses. We agree that future studies should aim for a more diverse study sample to enhance the generalizability of the results, and we have recommended this approach for future research in the manuscript (Page 15, lines 281-284).

**Reviewer comment 3:** MVPA Cutoff Limitations: The MVPA cutoff used was developed for healthy older adults and might underestimate the intensity of PA for those with more severe disabilities. This could lead to misclassification of activity intensity, especially for the LCA group.

**Authors response:** We agree that the MVPA cut-points used in our study, which were originally developed for healthy older adults, may indeed underestimate physical activity intensity for individuals with more severe disabilities post stroke, such as limited community ambulators. Ideally, cut-points specific to people with stroke—and tailored to different subgroups of high and low-functioning individuals post-stroke—would be available and used. However, since these specific cut-points do not yet exist, we used the established MVPA thresholds to ensure consistency with existing literature and facilitate comparability across studies.

This limitation was acknowledged in our manuscript. Despite these constraints, we believe that the use of the ActiGraph accelerometer remains justified, as it is recommended in a recent consensus paper (Fini et al., 2023).

We have also highlighted the need for the development and validation of physical activity intensity cut-points that more accurately reflect the physiological capabilities of individuals with different ambulation statuses post-stroke, and have included this as a recommendation for future research (Page 15, lines 290-293).

*How should we measure physical activity after stroke? An international consensus*

*Fini N, Simpson D, Moore S, Mahendran N, Eng J, Borschmann K, Moulaee Conradsson D, Chastin S, Churilov L, English C. Int J Stroke, 2023*

**Reviewer comment 4:** Lack of Longitudinal Perspective: The study is cross-sectional, limiting the ability to infer causality or observe changes in PA patterns over time.
**Authors response:** We acknowledge that the cross-sectional design and the absence of longitudinal data are limitations of this study. However, these aspects are beyond the scope of our research aims, which focused on providing insights into physical activity behavior in individuals post-stroke with varying ambulation statuses compared to healthy controls.

Despite these limitations, this study is significant because, to our knowledge, it is the first to analyze physical activity patterns in relation to different bout durations among people with diverse ambulation impairments. This unique perspective fills a gap in the literature and offers valuable information that can inform future research and interventions.

In the discussion section of the manuscript (Page 16, lines 297-301), we now underscore the importance of conducting longitudinal studies to examine levels and patterns of physical activity post-stroke. This research is essential for addressing key questions, such as understanding the long-term effects of physical activity on stroke recovery and cardiovascular health.

**Reviewer comment 5:** Methodological Differences: The paper mentions differences in accelerometer placement and PA bout definitions compared to previous studies, which may affect comparability.

**Authors response:** The methodology used in our study was tailored to address our specific research question and was carefully considered during the study design. We believe that this approach was well-suited for accurately capturing physical activity patterns in individuals post-stroke with varying ambulation statuses.

We agree with the reviewer that this is a crucial methodological consideration. Therefore, we have included a discussion in the manuscript (page 16, lines 295-297) highlighting the need for future research to investigate how accelerometer placement affects the accuracy of capturing various domains of physical activity post stroke, such as step counts and moderate-to-vigorous physical activity (MVPA).

**Reviewer comment 6:** Data Sharing Restrictions: The data availability statement indicates restrictions due to privacy concerns, limiting access for further validation and research.

**Authors response:** Since data can indirectly be traced back to the study participants, according to the Swedish and EU personal data sharing legislation, access can only be granted upon request from the Research Data Office at Karolinska Institute (es.ik@odr). Any sharing of data will be regulated via a data transfer and use agreement with the recipient and require ethical approval from the Regional Board of Ethics in Stockholm. This is the official practice of Karolinska Institutet that we are required to follow.

**Reviewer comment 7:** Lack of Severe Cases: The exclusion of individuals with severe ambulation impairments limits the study’s applicability to all stroke survivors.
**Authors response:** We acknowledge that the lack of individuals with severe ambulation impairments limits the generalizability of our findings to the broader stroke population. The inclusion of individuals with mild and moderate impairments was aligned with our research question. We have addressed this limitation in the discussion of our manuscript (page 15, lines 281-284) and recommended that future research should include a broader range of impairments to enhance the applicability of the findings.

In addition, a few minor comments
**Reviewer comment 8:** Language and Clarity: Some sections could benefit from clearer language to enhance readability.
**Authors response:** All authors have thoroughly reviewed the manuscript and made revisions to improve readability where appropriate. Please let us know if there are any additional language revisions needed.

**Reviewer comment 9:** Figures and Tables: Ensure all figures and tables are clearly labeled and referenced in the text for better comprehension.

**Authors response:** Thank you for this suggestion. We have clarified the references to Table 1 in the results section (page 8, line 180).

**Reviewer comment 10:** Data Presentation: More detailed presentation of the data analysis methods could improve transparency and reproducibility.
**Authors response:** We thank the reviewer for this comment; however, we believe we have provided sufficient information to ensure the study’s transparency and reproducibility. If the reviewer feels otherwise, we would appreciate more specific feedback on how we can improve the data presentation.

Overall, this paper is more suited for the audience in the specialist physiotherapy journal rather than PLOS ONE.

**Reviewer 2**

The results presented in the current report require a more thorough and detailed analysis to fully understand their implications. It is essential to provide a comprehensive explanation of what the values signify in the context of the study. This includes interpreting the numerical data and explaining how these values relate to the objectives and hypotheses of the research.

A detailed breakdown should include:

**Reviewer comment 1:** Contextual Interpretation: Elaborate on how the values fit within the broader context of the study. This means discussing the relevance of these results to the research questions and how they align with or diverge from expected outcomes.

**Authors response:** We are unclear about the intent of this comment. Additional clarification would be helpful for us to address it effectively.

**Reviewer comment 2:** Statistical Significance: Clarify the statistical significance of the results. Provide information on the confidence intervals, p-values, or other statistical metrics used to determine the reliability of the findings.
**Authors response:** Thank you for your comment. We report categorical data as counts and percentages, and use medians and interquartile ranges for continuous data. Group comparison was performed using chi-square, Fisher’s exact test for categorical data, whereas Mann–Whitney U-test and Kruskal-Wallis test was used for continuous data. We believe that this approach to reporting and statistical analysis is appropriate to the nature and distribution of the data.

**Reviewer comment 3:** Comparative Analysis: Compare the results with previous studies or benchmarks. Explain how these values contribute to or challenge existing knowledge in the field.
**Authors response:** Thank you for this comment. We do feel that we have compared our results with previous studies in this field sufficiently throughout the discussion section. Please let us know if there were specific aspects of the results that would benefit from a more detailed comparison with existing literature.

**Reviewer comment 4**: Practical Implications: Discuss the practical implications of the results. What do these values mean for practitioners, stakeholders, or policymakers? How can they be applied in real-world scenarios?
**Authors response:** The paper discusses the impact of this work on both the scientific community and clinical practice, emphasizing the importance of tailoring physical activity promotion strategies based on an individual's ambulation status. Practically, this means that strategies for encouraging physical activity should be customized to fit each person's level of mobility. Furthermore, the paper highlights the need for additional research to specifically address physical inactivity in individuals with limited ambulation following a stroke.
**Reviewer comment 5**: Visual Aids: Use graphs, charts, or tables to visually represent the data. This can help in making complex information more accessible and easier to understand.

**Authors response:** We have included two tables (Table I: Study characteristics, Table II: Physical activity bouts) and 1 figure (Figure 1: Daily number of physical activity bouts during the morning, afternoon and evening). Please let us know if there are specific aspects of the results that could benefit from additional visual representations or tables.
