# Supplementary material for: Characterizing physical activity bouts in people with stroke with different ambulation statuses
Source: PLoS One. 2025 May 14;20(5):e0307625. doi: 10.1371/journal.pone.0307625 (PMC12077672; doi:10.1371/journal.pone.0307625)
Supplement: Attachment — Submitted filename: Response to reviewer.docx [file pone.0307625.s003.docx]

## **Reviewer 3**

Firstly, I am grateful for the opportunity to review the manuscript entitled: Characterizing physical activity bouts in people with stroke with different ambulation statuses which aimed to characterize and contrast the daily patterns, frequency and intensity of physical activity bouts between people post stroke with different ambulation statuses compared to healthy controls.

Here are some aspects to consider in relation to your manuscript in order to increase the quality of your manuscript.

**Comment 1** Abstract.
In relation to the content of the abstract, it is important that the acronyms that appear are identifiable and that you have previously indicated what they refer to. It is especially suggested to pay attention to the acronyms LCA, CA, HC, MVPA, LIPA, which have not been previously indicated.
Such indication should also be considered in the manuscript.

***Author response*:** Thank you for bringing this concern to our attention. We acknowledge that the abstract and manuscript contain numerous abbreviations, which require clarification.

In the abstract, we have now clarified the abbreviations used for the two sub-groups of participants post-stroke:

- Limited community ambulators (LCA)
- Community ambulators (CA)

Additionally, to enhance clarity and minimize the number of abbreviations, we have decided to avoid using abbreviations for healthy controls, moderate-to-vigorous physical activity, and light physical activity in the abstract.

In the manuscript, we have now reviewed the use of abbreviations and made the following clarifications:

- We have now introduced CA and LCA in the second paragraph of the introduction (lines 55– 63).
- We have added definitions for LIPA and MVPA the first time these abbreviations appear in the introduction (lines 65-69).
- We have decided not to use the abbreviation for healthy controls (HC) to reduce the overall number of abbreviations.

**Comment 2.** Introduction.
It is suggested that the categorisation of the sample should be better defined, as the way it is expressed in the document may lead to confusion. In the case of the ‘sedentary exercisers’ group, it would be those who exercise 0.7 hours/day or more. Where are the subjects who exercise between 0.7 hours/day and < 0.5 hours/day included? In the case of the ‘sedentary movers’ group, does it correspond to those subjects who do <0.5hours/day and > 0.1 hours/day?
In the case of the ‘sedentary prolongers’ group, are they those who perform = or < 0.1 hours/day? It is really important that the categorisation of the sample is clearly stated in order to understand the results and the subsequent analysis and interpretation that follows.

***Author response*:** Thank you for your comment. The categorization the reviewer is referring to comes from a separate referenced study and is not used as a cutoff to classify the present study sample. Instead, we categorized individuals post-stroke based on their self-selected walking speed: limited community ambulators (LCA) (self-selected gait speed <0.8 m/s) and community ambulators (CA) (self-selected gait speed ≥0.8 m/s) (see method section lines 142–146).

To clarify this, we have introduced CA and LCA in the second paragraph of the introduction (lines 55–63) and removed the reference to the study mentioned by the reviewer to avoid confusion. Furthermore, in this paragraph, we have emphasized the relationship between mobility status, gait speed, and physical activity in individuals post-stroke.

To support this, we have added the following references:

- *Fini NA, Bernhardt J, Holland AEJD, rehabilitation. Low gait speed is associated with low physical activity and high sedentary time following stroke. 2021;43(14):2001-8.*
- *Bansal K, Fox EJ, Clark D, Fulk G, Rose DK. Speed- and Endurance-Based Classifications of Community Ambulation Post-Stroke Revisited: The Importance of Location in Walking Performance Measurement. Neurorehabil Neural Repair. 2024;38(8):582-94*.

Additionally, in the light of the revisions and clarifications made in the second paragraph regarding categorization of ambulation statuses, we have made minor revisions to the text in the fourth paragraph of the introduction where the CA and LCA are mentioned lines 79-80). Finally, we have revised the aims to emphasize the categorization of the study sample (lines 92–94).

**Comment 3.** Study participants.
In relation to the exclusion criteria, what assessment and scoring tools have been taken into account for the aspects of ‘cognitive impairments’, ‘severe neglect’ and ‘global aphasia’? It is recommended to provide such data to support the exclusion of participants.
Clinical assessments and performance-based clinical test
It is suggested that the authors provide the psychometric properties of the assessment tools used, as well as their validation and previous use in stroke survivors.

***Author response*:** We have now clarified (lines 107-110) that we used Montreal Cognitive Assessment (MoCA) to screen for cognitive impairments, Star Cancellation Test to screen for severe neglect and the ability to communicate through speech was evaluated informally during a telephone interview and while completing the MoCA. However, we did not use specific cut-off points for screening for e.g. cognitive impairments. Instead, we used these instruments to guide the screening process and make an informed decision about whether the person could participate in the test procedure in a safe and ethical manner, e.g., follow instructions during data collection.

Regarding the psychometric properties of the assessment tools used, we have now added references validating the performance-based clinical test for individuals post-stroke. The following references have been added to the manuscript:

*Chedoke-McMaster Stroke Assessment* (lines 125-126):

- *Gowland C, Stratford P, Ward M, Moreland J, Torresin W, Van Hullenaar S, et al. Measuring physical impairment and disability with the Chedoke-McMaster Stroke Assessment. Stroke. 1993;24(1):58-63*

*Fatigue Severity Scale* (line 129)*:*

- *Ozyemisci-Taskiran O, Batur EB, Yuksel S, Cengiz M, Karatas GK. Validity and reliability of fatigue severity scale in stroke. Top Stroke Rehabil. 2019;26(2):122-7.*

*Hospital Anxiety and Depression Scale (lines 133-135):*

- *Karlsson J, Hammarstrom E, Fogelkvist M, Lundqvist LO. Psychometric characteristics of the Hospital Anxiety and Depression Scale in stroke survivors of working age before and after inpatient rehabilitation. PLoS One. 2024;19(8):e0306754.*

*Montreal Cognitive Assessment* (lines 137-140):

- *Lau HY, Lin YH, Lin KC, Li YC, Yao G, Lin CY, et al. Reliability of the Montreal Cognitive Assessment in people with stroke. Int J Rehabil Res. 2024;47(1):46-51*.

**Comment 4** Assessment of physical activity.
It would be appreciated if the authors could provide a justification for the full 7-day monitoring with Actigraph GT3X+.

***Author response*:** Thank you for your comment. We have now added a justification for measuring physical activity over seven days in the manuscript (see lines 152–154).

We have also added a reference to support this decision:

- *Matthews CE, Hagstromer M, Pober DM, Bowles HR. Best practices for using physical activity monitors in population-based research. Med Sci Sports Exerc. 2012;44(1 Suppl 1):S68-76.*

**Comment 5.** Table 1.
It is suggested that the authors include the data in relation to the group of ‘healthy controls’.

***Author response*:** Thank you for this suggestion. In our manuscript, we have described the key characteristics of the healthy control group, specifically age and sex. Given that these are the only available variables for this group, we have opted not to present them separately in a table to maintain focus on the primary analyses.

**Comment 6.**
Discussion.
It would be appreciated to know why the use or non-use of assistive products has not been considered. On the other hand, it is suggested to provide such information in table 1.

***Author response*:** Thank you for the suggestion. We have now included information on the use of walking aids in Table 1. It was more common to use a walking aid among limited community ambulators (82%) than in the community ambulators (41%) subgroup, which is now also highlighted in the discussion (lines 265–266).

**Comment 7.** It is recommended to review the use of the term ‘basic activities of daily living’, it is mentioned 2 times in the discussion section. If it is linked to ‘walking’ it is important to reflect to which type of mobility it refers as well as which of them involve energy expenditure.
Please provide information about the sample in relation to the performance of these basic activities.

***Author response*:** We have reviewed the use of the term "basic activities of daily living" and have instead adopted the expression "low-intensity activities of everyday living", providing examples such as slow walking, household tasks, and gardening. These activities are commonly categorized as light-intensity physical activity (LIPA) but may represent moderate-to-vigorous physical activity (MVPA) for individuals with greater functional impairment. This distinction is now elaborated on in the discussion (lines 266–269).

**Comment 8.** References.
It is suggested that the authors update the references and include the most recent research and publications in both the introduction and the discussion.

***Author response*:** Thank you for this suggestion. We have updated the references across the manuscript and made the following revisions:

In the introduction, we refer to the preventive role of regular physical activity in reducing the risk of stroke-related disability and stroke recurrence (Hobeanu et al., 2022, lines 43–45). Additionally, we have added a recent study by Espernberger et al. (2025) on the diurnal patterns of physical activity post-stroke (lines 76–77). We have also added two recent references regarding the link between ambulation status and physical activity post-stroke (see our response to comment 2 above).

In the discussion, we have added a recent studies by Hall et al (2023) on the interventions for behaviour change and self-management of risk in stroke secondary prevention (line 284).

- *Hobeanu C et al. Risk of subsequent disabling or fatal stroke in patients with transient ischaemic attack or minor ischaemic stroke: an international, prospective cohort study. Lancet Neurol 2022*
- *Espernberger K et al. Physical activity patterns in independently mobile adult stroke survivors: an in-depth exploratory, observational study. Disabil Rehabil. 2025*
- *Hall P et al. Interventions for behaviour change and self-management of risk in stroke secondary prevention: an overview of reviews. Cerebrovasc Dis 2023*
